# Second‐trimester prenatal diagnosis of Nager syndrome with a deletion including *SF3B4* detected by chromosomal microarray

**DOI:** 10.1002/ccr3.2509

**Published:** 2020-02-06

**Authors:** Malgorzata Drozniewska, Mark D. Kilby, Julie Vogt, Fiona Togneri, Elizabeth Quinlan‐Jones, Lisa Reali, Stephanie Allen, Dominic McMullan

**Affiliations:** ^1^ West Midlands Regional Genetics Laboratory Birmingham Women’s & Children’s NHS Foundation Trust Birmingham UK; ^2^ Fetal Medicine Centre Birmingham Women's & Children’s NHS Foundation Trust Birmingham UK; ^3^ Birmingham Centre for Women's and New‐born Health College of Medical and Dental Sciences Institute of Metabolism and Systems Research University of Birmingham Birmingham UK; ^4^ Clinical Genetics Unit West Midlands Regional Genetics Service Birmingham Women’s & Children’s NHS Foundation Trust Birmingham UK

**Keywords:** microarrays, Nager syndrome, prenatal diagnosis, SF3B4 gene

## Abstract

Nager syndrome is a rare, complex malformation syndrome, for which there is limited information on prenatal genetic testing. Clinical diagnosis of Nager syndrome, which can be caused by deletions encompassing SF3B4 gene, is possible prenatally. Prenatal chromosomal microarray can aid genotype‐phenotype correlation in pregnancies with structural abnormalities seen on ultrasound.

## Graphical abstract

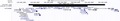

## 1 INTRODUCTION

Acrofacial dysostoses (AFDs) are a clinically heterogeneous group of disorders. Two major groups have been distinguished using postnatal clinical features, dependant on the type of limb defect: predominant preaxial defects (including Nager syndrome) and postaxial malformations (including Genee‐Wiedemann or Miller syndromes).^1^, ^2^ Preaxial abnormalities are caused by abnormal development of the first and second branchial arches and limb buds.^3^

Nager syndrome (NS), first described by Nager and de Reyenie in 1948 (MIM #154400), is the most common form of AFD (also called mandibulofacial dysostosis or acrofacial dysostosis type 1; AFD1). It is a rare, complex malformation syndrome, characterized by craniofacial findings (micrognathia, cleft palate, ear anomalies) and limb malformations (hypoplasia or absent thumbs, radioulnar dysostosis, short forearm).^3^, ^4^, ^5^ Other clinical features in childhood may include short stature, renal or cardiac anomalies, and costovertebral defects.^5^

There are less than 100 reported cases of NS, only 10 of which have been suspected prenatally.^1^, ^4^, ^5^, ^6^, ^7^

Several probands have been diagnosed in the second or third trimester using a detailed ultrasound assessment often with suspected hypoplasia of the mandible and facial anomalies such as micrognathia and midface retrusion. In addition, limb anomalies have been described. In cases which have been diagnosed postnatally, micrognathia with associated airway obstruction may be severe enough to require tracheostomy in early childhood and approximately 1:5 affected children die in the newborn period.^6^, ^8^ Developmental morbidity may be variably present.^9^

Although mostly sporadic, both autosomal dominant and recessive inheritance of NS have been reported, suggesting genetic heterogeneity of this condition.^5^, ^8^ Heterozygous mutations in the *SF3B4* gene (1q21.2) cause clinical NS in approximately 56% of cases,^8^ and 32 distinct *SF3B4* mutations have been identified (in 38 unrelated patients). This includes 5 prenatally suspected cases diagnosed using ultrasound, with subsequent genetic confirmation after birth or at termination of pregnancy (TOP). All reported mutations are predicted to cause loss of function of *SF3B4*. Whole‐gene deletion has also been associated with NS.^7^

*SF3B4* (OMIM *605593, Splicing Factor 3B, Subunit 4) has been mapped to chromosome 1. It encodes SAP49, a spliceosome‐associated protein, belonging to the group of seven proteins in the mammalian SF3B complex. SAP49 plays a key role in RNA splicing by binding to the pre‐mRNA and interacting with other U2 snRNPs which results in tethering the U2 snRNP to the branch site.^5^, ^8^ In addition, SAP49 is linked with bone morphogenic protein (BMP) signaling. This pathway is involved in early embryogenesis and skeletal development, and it has been demonstrated that SAP49 specifically inhibits BMP‐mediated osteochondral cell differentiation. In mouse embryos, *SF3B4* is expressed at a high level in forelimbs, hind limbs, and somites during early stages suggesting its involvement in skeletal development. *SF3B4* displays a dynamic pattern of expression in the developing heart as well.^5^

## 2 CASE STUDY

A 21‐year‐old Caucasian woman, with a previous first‐trimester miscarriage, was referred to the Fetal Medicine Centre for her current pregnancy because of an increased first‐trimester combined screening risk (risk of trisomy 21/trisomy 18 and 13 at 1:5) and an enlarged nuchal translucency of 4.5mm at 12 weeks and 5 days gestation (based on fetal crown‐rump length). The patient had a body mass index of 23, and there was no reported consanguinity.

At 13 weeks and 1 day, a detailed transabdominal ultrasound scan (USS) was performed. This demonstrated a viable singleton pregnancy and confirmed an increased nuchal translucency measurement greater than the 99% confidence interval for crown‐rump length (CRL). The fetal legs were abnormally extended bilaterally with measured femoral, tibial, and fibular lengths of less than the 5th centile for gestation. There were also severe bilateral talipes equinovarus and an abnormal‐shaped fetal skull (“strawberry” appearance).

The patient was initially reluctant to undergo prenatal testing by chorionic villus sampling due to her previous miscarriage. However at 15 weeks, gestation gave consent for an amniocentesis. At this time, a further detailed USS was performed again noting that the bones of the lower limbs were significantly foreshortened with associated leg “fixed” extension of the knees and severe bilateral talipes. Again, the fetal skull was noted to be of an abnormal shape. The four‐chamber view and outflow tracts appeared normal.

An uncomplicated amniocentesis was performed and 20 mL of clear amniotic fluid sent for quantitative fluorescent PCR (QF‐PCR) and chromosomal microarray analysis. Parental blood samples were also taken.

QF‐PCR demonstrated no evidence of trisomy 13, 18, or 21 and no evidence of sex chromosome aneuploidy.

**Figure 1:**
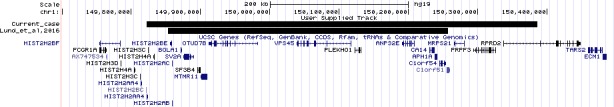
Comparison of current case with the case reported by Lund et al, 2016. UCSC genome browser on Human Feb. 2009 (GRCh37/hg19) Assembly

Chromosomal microarray analysis (CMA) was performed using the BlueGnome 8x60k v2.0 ISCA platform. Test DNA was referenced against same‐sex control DNA, and data were analyzed in BlueFuse Multi v4.1. The analysis revealed a male profile with a de novo deletion of approximately 565kb at chromosome 1q21.2 (arr [hg19] 1q21.2 (149821840_150386661) x1 dn). The region of imbalance included 21 protein‐coding genes, including *SF3B4* (Figure 1). None of the other genes are currently associated with the presenting clinical features, although notably 4 genes (*ANP32E*, *PLEKHO1*, *PRPF3,* and *RPRD2*) have pLI scores (indicating that a gene is intolerant to a loss of function (LoF) mutation) higher than 0.9, suggesting haploinsufficiency for these genes may not be tolerated.^10^

A further scan was performed at 18 weeks and 1‐day gestation, and similar findings were noted. The femur, tibia, and fibula were all <1st centile for gestation. A cleft lip was identified but the palate was not visualized, and there was a suspicion of micrognathia. Again, severe bilateral talipes was observed.

After detailed discussion with the Clinical Genetics Team, this couple underwent a TOP. They declined postmortem examination of the fetus.

## 3 DISCUSSION

*SF3B4* was reported as a strong candidate gene for Nager type of acrofacial dysostosis by Bernier et al after exome sequencing of 35 families with a clinical diagnosis of NS. Nonsense, frameshift, and splice site mutations have been identified, and it has been suggested that NS is a result of haploinsufficiency of *SF3B4*.^8^ This was confirmed by Petit et al after exome sequencing of 14 families.^5^ Whole‐gene deletions are rare findings, and to the best of our knowledge, this is only the second reported case of a whole *SF3B4* deletion. The first reported prenatal case by Lund et al was a de novo 0.4 Mb deletion encompassing 15 refseq genes, including *SF3B4*.^7^ Clinical and genetic details of the current case and cases reported previously are summarized in Table 1.

**Table 1:** Comparison of clinical and genetic details in current case and cases reported previously

	Current case	Lund et al^7^	Castori et al^4^	McPherson et al^6^	Petit et al^5^	Ansart‐Franquet et al^1^
GA at diagnosis (wks)	13 + 1	12	22	22	28	22
Ultrasound features						
Enlarged NT	+					
Micrognathia/hypoplastic mandible	+	+	+	+	+	+
Preaxial upper limb anomalies		+	+		+	+
Upper limb anomalies: both “rays”		+		+		
Club foot/feet	+	+		+		
Absent fibula				+		
Foreshorten long bones	+	+		+		
Diaphragmatic hernia			+			
Cardiac anomaly	+ (LV echogenic focus and TR)					
HC	Normal	Normal	N/A	Normal	+1sd	Twin 1: −1sd Twin 2: −2sd General SGA
Abnormal head shape	+					
Sample	Amniocentesis	CVS	POC	Amniocentesis	POC	PM: lung tissue
SF3B4 mutation and/or chromosomal anomaly	arr[hg19] 1q21.2(149821840_150386661)x1 dn	arr[hg19] 1q12.2(149852674_150257430) x1 dn	SF3B4: c.35‐2A > G	SF3B4: c.164‐1G > A	SF3B4: c1060dupC	SF3B4: c1A > G
Outcome	TOP	TOP	TOP	Live born	TOP	TOP

Abbreviations: CVS, chorionic villus sampling; GA, gestational age; HC, head circumference; LV, left ventricle; N/A, not applicable; NT, nuchal translucency; PM, postmortem; POC, products of conception; Sd, standard deviation; SGA, small for gestational age; TOP, termination of pregnancy; TR, tricuspid regurgitation.

With the more widespread use of high‐resolution chromosomal microarray testing in pregnancies where developmental abnormalities have been detected on USS, it should be expected that more case studies will be presented in which NS is diagnosed prenatally in the first trimester and associated with whole‐gene deletion of *SF3B4*.

This prenatally diagnosed case further supports previous findings that alterations and whole‐gene deletions including *SF3B4* cause clinical features of NS. Furthermore, it demonstrates the importance and clinical usefulness of chromosomal microarray analysis in prenatal settings for early diagnosis and confirmation of the underlying cause of antenatally detected fetal anomalies.

## CONFLICT OF INTEREST

None declared.

## AUTHOR CONTRIBUTIONS

[MD]: made substantial contribution to conception and design and analysis and interpretation of data. [FT], [LR], [SA], and [DM]: made substantial contribution to analysis and interpretation of data. [MDK] and [JV]: made substantial contribution to data acquisition. [EQ‐J]: contributed to manuscript preparation. All authors were involved in revising the manuscript critically for important intellectual content. All authors read and accepted the final manuscript.

## References

[1] Ansart‐Franquet H , Houfflin‐Debarge V , Ghoumid J , et al. Prenatal diagnosis of Nager syndrome in a monochorionic‐diamniotic twin pregnancy. Prenat Diagn. 2009;29:187‐189. 19180573 10.1002/pd.2214

[2] Rios LT , Araujo Júnior E , Nardozza LM , et al. Prenatal diagnosis of Nager syndrome in the third trimester of pregnancy and anatomopathological correlation. J Med Ultrason. 2012;39:287‐289. 10.1007/s10396-012-0374-7 27279120

[3] Lin JL . Nager syndrome: a case report. Pediatr Neonatol. 2012;53:147‐150. 22503264 10.1016/j.pedneo.2012.01.014

[4] Castori M , Borttillo I , D'Angelantonio D , et al. A 22‐week‐old fetus with Nager syndrome and a congenital diaphragmatic hernia due to a novel SF3B4 mutation. Mol Syndromol. 2014;5:241‐244. 25337072 10.1159/000365769 PMC4188155

[5] Petit F , Escande F , Jourdain AS , et al. Nager syndrome: confirmation of SF3B4 haploinsufficiency as the major cause. Clin Genet. 2014;86:246‐251. 24003905 10.1111/cge.12259

[6] McPherson E , Zaleski C , Ye Z , Lin S . Rodriguez syndrome with SF3B4 mutation: a severe form of Nager syndrome? Am J Med Genet. 2014;164A:1841‐1845. 24715698 10.1002/ajmg.a.36555

[7] Lund ICB , Vestergaard EM , Christensen R , et al. Prenatal diagnosis of Nager sundrome in a 12‐week‐old fetus with a whole gene deletion of SF3B4 by chromosomal microarray. Eur J Med Genet. 2016;59:48‐50. 26679067 10.1016/j.ejmg.2015.12.001

[8] Bernier FP , Caluseriu O , Ng S , et al. Haploinsuffiency of *SF3B4*, a component of the pre‐mRNA spliceosomal complex, causes Nager syndrome. Am J Hum Genet. 2012;90:925‐933. 22541558 10.1016/j.ajhg.2012.04.004 PMC3376638

[9] Czeschik JC , Voigt C , Alanay Y , et al. Clinical and mutation data in 12 patients with the clinical diagnosis of Nager syndrome. Hum Genet. 2013;132:885‐898. 23568615 10.1007/s00439-013-1295-2

[10] Lek M , Karczewski KJ , Minikel EV , et al. Analysis of protein‐coding genetic variation in 60,706 humans. Nature. 2016;536:285‐291. 27535533 10.1038/nature19057 PMC5018207

